# Mutational Landscape and Environmental Effects in Bladder Cancer

**DOI:** 10.3390/ijms21176072

**Published:** 2020-08-23

**Authors:** Takuji Hayashi, Kazutoshi Fujita, Yujiro Hayashi, Koji Hatano, Atsunari Kawashima, David J. McConkey, Norio Nonomura

**Affiliations:** 1 Department of Urology, Graduate School of Medicine, Osaka University, 2-2 Yamada-oka, Suita, Osaka 565-0871, Japan; takujihayashi0929@gmail.com (T.H.); yujiro8840103@gmail.com (Y.H.); koj.hatan@gmail.com (K.H.); kawashima@uro.med.osaka-u.ac.jp (A.K.); nono@uro.med.osaka-u.ac.jp (N.N.); 2 Greenberg Bladder Cancer Institute, Johns Hopkins University School of Medicine, Baltimore, MA 21287-2101, USA; dmcconk1@jhmi.edu; 3 Department of Urology, Faculty of Medicine, Kindai University, Ohno-higashi, Osakasayama, Osaka 589-8511, Japan

**Keywords:** bladder cancer, mutation, smoking, lifestyle, diet, inflammation

## Abstract

Bladder cancer is the most common cancer of the urinary tract. Although nonmuscle-invasive bladder cancers have a good prognosis, muscle-invasive bladder cancers promote metastases and have a poor prognosis. Comprehensive analyses using RNA sequence of clinical tumor samples in bladder cancer have been reported. These reports implicated the candidate genes and pathways that play important roles in carcinogenesis and/or progression of bladder cancer. Further investigations for the function of each mutation are warranted. There is suggestive evidence for several environmental factors as risk factors of bladder cancer. Environmental factors such as cigarette smoking, exposure to chemicals and gases, bladder inflammation due to microbial and parasitic infections, diet, and nutrition could induce several genetic mutations and alter the tumor microenvironment, such as immune cells and fibroblasts. The detailed mechanism of how these environmental factors induce carcinogenesis and/or progression of bladder cancer remains unclear. To identify the relationship between the mutations and the lifestyle could be useful for prevention and treatment of bladder cancer.

## 1. Introduction

Bladder cancer is the most common cancer of the urinary tract with 430,000 new cases and 165,000 deaths per year worldwide [1]. At diagnosis, the majority of bladder cancers are nonmuscle-invasive papillary tumors of low grade, which are termed as nonmuscle-invasive bladder cancer (NMIBC). NMIBC includes stage Ta, T1 tumors and carcinoma in situ (CIS). NMIBCs frequently recur (50–70%) but infrequently progress to invasion (10–15%) and have a good prognosis [2]. High-grade papillary tumors and flat dysplastic lesions, designated CIS, may progress to muscle-invasive bladder cancer (MIBC). MIBCs (of stage T2 and above) have less favorable prognosis, with five-year survival <50% and common progression to metastasis [3]. Although men are more likely to develop bladder cancer, women often present with more advanced disease and have unfavorable prognosis [4].

Comprehensive analyses using RNA sequence of clinical tumor samples in NMIBCs and MIBCs have been reported [5,6,7,8,9,10]. These analyses revealed that MIBCs have heterogeneity and can be divided into several molecular subtypes. At the highest level, MIBCs could be divided into basal and luminal subtypes [11]. These reports implicated the candidate genes and pathways that play important roles in carcinogenesis and/or progression of bladder cancer. DNA mutational patterns of bladder cancer were identified that are predominantly comprised of APOBEC (apolipoprotein B mRNA editing enzyme catalytic polypeptide) mutational signatures. Epithelial-to-mesenchymal transition (EMT), a process increasing invasion and migration, characterized by loss of homotypic adhesion and cell polarity, is one of the key factors for drug sensitivity and metastasis in bladder cancer [12].

Age, gender, cigarette smoking, exposures to chemicals and gases, certain medications, radiation, and genetic factors are established risk factors for initiation and progression of bladder cancer. There is suggestive evidence for several other environmental factors including diet, nutrition, and metabolic syndrome [13,14]. Inflammation is one of the key factors for initiation and/or progression of various types of cancer. Lifestyle, especially dietary habits, is the basis of chronic systemic inflammation, which also constitutes a risk for diabetes mellitus, cardiovascular disease, neurodegenerative diseases, and certain cancers including breast, colon, prostate, and pancreatic cancer [15,16]. Several epidemiological studies about association of pro-inflammatory diet with bladder cancer have been reported [17]. Environmental factors could induce several genetic mutations as smoking damages DNA and reduces repair activity [18]. The mechanism of how environmental factors induce carcinogenesis and progression of bladder cancer remains unclear.

**Figure 1:**
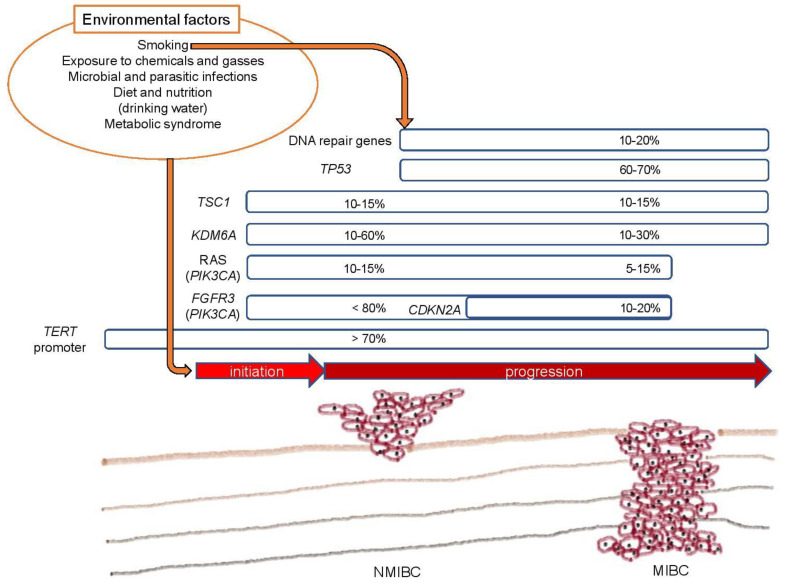
An overview of mutational landscape and environmental effects in bladder cancer. NMIBC, nonmuscle-invasive bladder cancer; MIBC, muscle-invasive bladder cancer.

In this review, we discuss the representative genetic mutations in bladder cancer and the potential effects of environmental factors on initiation and/or progression of bladder cancer (Figure 1).

## 2. Representative Genetic Mutations in Bladder Cancer

The results of comprehensive analyses using RNA sequence revealed several representative genetic mutations in bladder cancer. These mutations suggested candidate genes that are key factors for carcinogenesis and/or progression from NMIBC to MIBC.

### 2.1. TERT Promoter

Activating mutations in the promoter of the telomerase reverse transcriptase gene *TERT* lead to elevated telomerase expression and allow several cancers to overcome the end-replication problem and avoid senescence. Mutations in *TERT* promoter are the most frequent events identified in bladder cancers of all grades and stages. More than 70% of Ta and T1 tumors have mutations, largely confined to two hotspot nucleotides at positions −124 bp (base pair) and −146 bp upstream from the ATG translation initiation codon [5]. The mutations have also been found in 60% of CIS samples [19], suggesting that this is a universal and early event in the development of both NMIBC and MIBC. While the mutation of *TERT* promoter is an early event in bladder carcinogenesis, hypermethylation of *TERT* promoter is a dynamic process that contributes to disease progression [20].

These mutations are identified in both the normal tissues, which may be precancerous lesion, and the tumor samples of bladder cancer including various rare histological variants (micropapillary, plasmacytoid, adenocarcinoma, squamous cell carcinoma) [21,22,23,24,25,26]. These mutations can also be detected with ease in both the pellet and cell-free DNA from patient urine samples, promising application in urine-based disease detection and prediction of progression [27,28,29,30].

### 2.2. FGFR3

Fibroblast growth factor receptor, *FGFR3,* point mutation is more common in Ta (up to 80%) than in T1 (10–30%) and MIBC (10–20%). In MIBC, most of the tumors having the mutations are included in a subgroup of luminal subtype, luminal-papillary subtype [31]. The mutations are located in several hotspots, with the most common generating novel cysteine residues that are predicted to drive ligand-independent dimerization. FGFR3 can also be activated by the generation of fusion proteins that retain the kinase domain of the gene fused with another gene, commonly *TACC3* [32].

FGFR inhibitors have been evaluated in clinical trials [33]. It was reported that erdafitinib, one of tyrosine kinase inhibitors of FGFR1–4, had strong clinical activity in locally advanced and metastatic bladder cancer with *FGFR* alterations [34].

### 2.3. RAS Gene (KRAS or HRAS)

RAS gene (*KRAS* or *HRAS* oncogene) mutations are found in 10–15% of NMIBC and mutually exclusive with *FGFR3* mutations. Ras superfamily of monomeric G proteins participates in bladder cancer progression with other molecules such as epidermal growth factor receptor, p53, and PTEN (phosphatase and tensin homolog) [35]. Multiple components of the Ras-MAPK (mitogen-activated protein kinase) pathway have been identified as potential targets for bladder cancer [36].

### 2.4. PIK3CA

*PIK3CA* is activated by point mutation at higher frequency in NMIBC than in MIBC. *PIK3CA* mutations are most commonly found with *FGFR3* or RAS mutations. The *PIK3CA* mutation spectrum in bladder cancer differs significantly from that in other cancers. Mutations E542K and E545K in the helical domain are most common and the kinase domain mutation, H1047R, which is the most common mutation in other cancers, is less common in bladder cancer [37]. PIK3CA have domains for binding to RAS. There may be cooperation between PIK3CA mutant proteins and other events that are known to activate RAS [37].

### 2.5. KDM6A

*KDM6A* (Lysine (K)-specific demethylase 6A, H3K27 (Lysine 27 on histone H3) demethylase), a histone modifier, is commonly mutated in both NMIBC and MIBC (among all molecular subtypes). Loss of KDM6A function is expected to lead to gene silencing via transcriptional repression. *KDM6A* is located on the X-chromosome and both alleles are transcribed in females [38]. In contrast to MIBC, a strong gender bias that more mutations of *KDM6A* were present in females than males was observed in NMIBC [39]. Approximately half of the mutations were accompanied by loss of the second gene copy or were co-heterozygotic. Although bladder cancer is much more prevalent in males, female patients have a worse prognosis [4]. This gender difference persists even after correction for environmental factors such as smoking. While it has been ascribed to differences in sex hormones, particularly androgens, it could also result from the protective effect of two functional *KDM6A* copies in females.

The study using *KDM6A*-knockout bladder cancer cells and patient-derived xenograft model suggested that EZH2 (enhancer of zeste homolog 2, H3K27 methylase) inhibition is a potential therapeutic target for bladder cancer with the mutations [40]. In the recent study using the mice lacking *Kdm6a* in the urothelium, *Kdm6a* deficiency activates inflammatory pathways, promotes M2 macrophage polarization, and causes bladder cancer in cooperation with *p53* dysfunction [41].

### 2.6. TSC1

Chromosome 9 deletions are found in more than 50% of bladder tumors of all grades and stages. In NMIBC, loss of the long arm (9q) is most common. Mutations of *TSC1* (tuberous sclerosis 1, 9q34) are found in 10–15% of bladder cancers with no clear association with grade or stage. *TSC1* is one of two genes that, when mutated in the germline, cause the syndrome tuberous sclerosis complex, an autosomal-dominant, tumor-suppressor gene syndrome characterized by the development of hamartomas in the kidneys, heart, brain, and skin.

Missense mutations of *TSC1* found in bladder cancer were shown to cause loss of function through aberrant splicing, protein instability, or protein mislocalisation [42]. Loss of TSC1 leads to hypoacetylation of Heat shock protein 90 (Hsp90) and subsequent decreased binding to the Hsp90 inhibitor [43]. Combined Hsp90/mTOR (mammalian target of rapamycin) inhibition is a promising therapeutic approach for *TSC1*-mutant bladder cancer [44].

### 2.7. CDKN2A

Loss of the short arm (9p) is found at lower frequency than loss of 9q in NMIBC. The 9p deletions are focused on *CDKN2A* (cyclin-dependent kinase inhibitor 2A) locus (encoding p16 and p14^ARF^ (ADP (adenosine diphosphate) -ribosylation factor). Loss of *CDKN2A* is implicated to play an important role in progression of *FGFR3*-mutant NMIBC to MIBC [45]. In the TCGA (The Cancer Genome Atlas) data, *CDKN2A* expression was significantly higher in basal subtype than in luminal subtype [46]. MIBC patients with high expression of *CDKN2A* have poor prognosis. Further investigations into the function and potential for biomarkers of *CDKN2A* are warranted in both NMIBC and MIBC.

### 2.8. TP53

Mutation frequency of *TP53*, which is one of major tumor suppressor genes, is very low in low-grade Ta but higher in T1 and MIBC. *TP53* mutations are considered an early event in the development of CIS lesions. Inactivation of p53 may lead to increased propensity of CIS lesions to progress to invasive tumors, compared to other superficial tumors [47]. A p53-like molecular subtype of MIBC characterized by wild-type p53 gene expression signatures shows primary and acquired resistance to chemotherapy [7].

*TP53* mutations were enriched in tumors with genome-doubling events, suggesting that loss of *TP53* activity facilitates genome doubling [48]. The oncogene MDM2 (murine double minute 2) protein inhibits p53 transactivation function by binding to a region of the *TP53* transactivation domain. The p53 gene family members p53, p73, and p63 display several isoforms derived from the presence of internal promoters and alternative splicing events. Several agents targeting p53 pathway such as synthetic peptides derived from the p53 C-terminal domain have been developed for the treatment of bladder cancer.

The heterozygous p53 knockout mice develop tumors within six months of exposure to genotoxic carcinogens. P53 function does not affect cytotoxicity or cell proliferation induced by *p*-cresidine, bladder carcinogen [49]. P53 involvement in mouse tumorigenesis may be more important in facilitating the malignant progression of neoplastic foci rather than in the initiation or promotion stage.

### 2.9. DNA Repair Genes (ERCC2, ATM, ATR, BRCA1, BRCA2, POLE, and FANCA)

Several DNA repair genes (*ERCC2*, *ATM*, *ATR*, *BRCA1*, *BRCA2*, *POLE*, and *FANCA*) are more frequently mutated in high-grade NMIBC and MIBC [5].

*ERCC2* (excision repair cross-complementation group 2) is a DNA helicase and a member of the nucleotide excision repair pathway, which repairs intrastrand crosslinks created by genotoxins such as UV irradiation and platinum chemotherapies [50]. In two cohorts of cisplatin-based chemotherapy in MIBC, patients with *ERCC2*-mutated tumors had improved survival compared to those with *ERCC2* wild-type tumors [51,52]. Feki-Tounsi M. et al. showed that slight protective effect of polymorphism of *ERCC2* codon 751 Gln/Gln genotype increased with age in a case-control study [53]. Additionally, the polymorphism seems to reduce bladder cancer risk among smoker and/or alcohol consumers. Further studies about the relation of smoking with the function of *ERCC2* are needed.

### 2.10. Others

*STAG2* (stromal antigen 2), a subunit of cohesion, was significantly and commonly mutated or lost in bladder cancer, mainly in tumors of low stage or grade, and its loss was associated with improved outcome [54].

*PTEN* and *FOXA1* are downregulated by allelic loss and site-specific DNA hypermethylation, respectively. Conditional inactivation of both *Pten* and *Foxa1* in intermediate/luminal cells in mice resulted in development of bladder cancer exhibiting squamous features, which suggested that hypermethylation of *FOXA1* and allelic loss of *PTEN* drives squamous differentiation and promotes heterogeneity [55].

## 3. Potential Effects of Environmental Factors on Bladder Cancer

Cigarette smoking, exposures to chemicals and gases, certain medications, radiation, and genetic factors are established risk factors for initiation and progression of bladder cancer. There is suggestive evidence for several other environmental factors including diet, nutrition, and metabolic syndrome. Environmental factors could induce several genetic mutations and alter the tumor microenvironment such as immune cells and fibroblasts.

### 3.1. Smoking

Cigarette smoking is the major environmental risk factors for bladder cancer. It is estimated that almost half of male patients and a quarter of female patients of bladder cancer can be attributed to smoking [56].

ERCC2 signature mutations were at higher levels in smokers than nonsmokers [30]. Fantoni F. et al. reported molecular footprints of MIBCs in smoking and nonsmoking patients [57]. In the TCGA data, only *GRP15* expression was significantly upregulated in smokers (independent from luminal and basal subtypes). *GRP15* is an orphan G protein-coupled receptor expressed by lymphocytes and mediates recruitment of effector T cells to inflamed tissue. *FLG*, *SPTAN1*, *USH2A*, *LYST*, and *MED13* were more frequently mutated in nonsmokers, whereas *SPTA1* was more frequently mutated in smokers. *TP53*, *TIN*, *KDM6A*, *MLL2* (*KMT2D*), and *MUC16* were the most frequently mutated genes in smokers. Mutational signatures of smokers were similar to those of BBN (N-butyl-N-(4-hydroxybutyl) nitrosamine) mouse model, where tumors are induced by a nitrosamine compound related to the carcinogens found in smoke. This model is known to have similarity with basal subtype [58]. Using this model, the methods to detect bladder cancer in early stage are developed [59]. Analyses of gene expression and mutations identified only a limited set of differences between smokers and nonsmokers, suggesting that tumors originating as the consequence of smoking or other causes can progress in a similar fashion, resulting in similar transcriptional defects.

Exposure of human normal urothelial cells to smoke induced morphological change, along with EMT and MAPK activation [60]. ERK1/2 and p38 inhibitors attenuated smoke-triggered urocytic EMT. Cigarette smoke extract exposure induced morphological change of human bladder cancer cells and enhanced EMT via activation of ERK1/2 pathway [61]. These results suggest that cigarette smoke induces initiation and progression of bladder cancer, mediating EMT and activating ERK1/2 pathway. Kispert S. et al. reported that the exposure of bladder cancer cells to cigarette smoke extract results in increased platelet-activating factor accumulation and increased expression of the platelet-activating factor receptor [62].

### 3.2. Exposure to Chemicals and Gases

It has been reported that there is occupational carcinogen exposure as a part of risk factors of bladder cancer. Agents with a suspected/established role as occupational bladder carcinogen include 2-naphthylamine, 4-aminobiphenyl, toluene, 4,4′-methylenebis (2-chloroaniline), metal-working fluids, polyaromatic hydrocarbons, perchloroethylene, and diesel exhaust [63]. The detailed mechanism by which each carcinogen induces bladder cancer remains unclear.

In order to discriminate carcinogens and noncarcinogens, in vitro genotoxicity tests are considered useful tools [64]. Isothiocyanates are highly biologically active compounds formed upon enzymatic hydrolysis of glucosinolates, naturally occurring thioglycosides contained in a variety of cruciferous vegetables. Although these compounds are effective chemopreventive agents against the carcinogenic effect of different compounds, including nitrosamines and polycyclic hydrocarbons, the results of the test for genotoxic effects indicated that the compounds are genotoxic, and probably carcinogenic [65]. Reactive oxygen species might be involved in the genotoxic effect of the isothiocynates.

### 3.3. Bladder Inflammation Due to Microbial and Parasitic Infections

Chronic local inflammation by recurrent urinary tract infections is associated with an increased bladder cancer risk, particularly squamous cell carcinoma [66,67]. The association between bladder schistosomiasis infections, inflammation, and bladder cancer risk has been well established [63]. It was reported that several urinary taxa such as *Fusobacterium*, *Sphingobacterium*, and *Enterococcus* distinguished urogenital schistosomiasis-induced bladder pathologies from urogenital schistosomiasis infection alone and from healthy persons [68]. Strains of bacteria that can mediate the formation of N-nitrosamines have been proposed to contribute to schistosomiasis-induced bladder cancer as well [69].

There is a hypothesis that urinary microbiome may alter the extracellular matrix, which may promote or inhibit urothelial carcinogenesis [70]. The association of genitourinary cancer including bladder cancer with urinary microbiome has been investigated [71].

From microbe analysis in the TCGA data, viral infection such as human papilloma virus and polyoma virus may contribute to a small percentage of bladder cancer [31].

### 3.4. Diet and Nutrition

#### 3.4.1. Pro-Inflammatory Diet

Diet can induce systemic and/or local inflammation. The association between the inflammatory potential of diet and the risk of urologic cancer including bladder cancer has been investigated in a small number of studies [18]. Dietary inflammatory index (DII), which is computed based on dietary intake assessed using a reproducible and valid 80-item food frequency questionnaire, is used as the indication of pro-inflammatory diet. Although one case-control study demonstrated the positive relation of the diet with the risk of bladder cancer [72], three prospective cohort studies showed no relation [73,74,75].

A case-control study of 670 bladder cancer cases and 665 controls demonstrated that the highest quartile of DII scores had twofold excess risk of bladder cancer compared to the lowest quartile [72]. There are possibilities of recall, selection, and reverse causation bias as well as incomplete control of confounding in this study. In a prospective cohort of more than 41,000 participants, a diet with pro-inflammatory potential was not associated with the overall bladder cancer risk [73]. In a prospective cohort study with 172,802 women and 45,272 men in the United States, high empirical dietary inflammatory pattern scores were not associated with a higher risk of bladder cancer (RR (relative ratio) 0.92, 95% CI (confidence interval) 0.75–1.12, P_trend_ (P-value for trend analysis) 0.67) [74]. These results were similar regardless of smoking status. In a prospective cohort with 101,721 participants, Luo J. et al. reported that energy-adjusted DII scores were not associated with bladder cancer risk in the multivariate models [75]. The HRs (hazard ratios) (95% CIs) of one-unit increment were 0.99 (0.96–1.02) and 1.01 (0.94–1.10) for men and women, respectively. It was suggested that local rather than systemic inflammation might have a role in the etiology of bladder cancer.

Dietary cholesterol was positively associated with the risk of various types of cancer including bladder cancer in a case-control study [76]. In another case-control study, statistically significant reduced odds of bladder cancer were observed for high intakes (highest quartile vs. lowest quartile) of α-linolenic acid (OR (odds ratio) 0.26, 95% CI 0.19–0.98, P_trend_ 0.01) and vegetable fat (OR 0.39, 95% CI 0.18–0.86, P_trend_ 0.06) [77].

#### 3.4.2. Fruit and Vegetable

Overall fruit and vegetable intake showed a significant protective effect on bladder cancer (RR 0.81, 95% CI 0.67–0.99) in a meta-analysis [78]. The dose–response analysis demonstrated that the risk of bladder cancer decreased by 9% (RR 0.91, 95% CI 0.83–0.99) and 8% (RR 0.92, 95% CI 0.87–0.97) for every 200 g/day increment in fruit and vegetable consumption, respectively [79]. A case-control study showed that the protective effect of vegetable consumption, especially cruciferous vegetables, may be modified by genetic variants of *GSTM1* and *NAT2*, carcinogen-detoxification genes [80]. Further well-designed prospective studies are warranted to confirm these findings.

Dietary açai fruit pulp reduced bladder cancer incidence, tumor cell proliferation, and p63 expression in mice, probably due to its potential antioxidant action [81]. Açai pulp presented a significant reduction in DNA damage induced by H_2_O_2_, a notable oxidant agent.

#### 3.4.3. Others (Alcohol, Coffee, Arsenic, Drinking Water, Meat, Vitamins)

Although those who reported high alcohol intake have an increased risk of bladder cancer, there was no dose response. These results suggest possible confounding lifestyle factors [63].

Current evidence remains equivocal whether coffee consumption may be associated with risk of bladder cancer. Some analyses showed positive association [82,83,84], while other analyses demonstrated inverse association [85,86]. Some reports concluded that no statistically significant association was observed [13,87]. A prospective study revealed that positive association between coffee and bladder cancer was attenuated after adjustment for smoking and other potential confounders [84]. In an international, pooled study, positive association was observed in different subgroups, only in never smokers [88] or only in male smokers [89]. These conflicting results suggest that the association between coffee and bladder cancer may be affected by gender and smoking status.

The exposure to arsenic in drinking water is a recognized cause of bladder cancer. A systematic review and a meta-analysis showed a risk effect of 2.7 (95% CI 1.2–4.1) for 10 μg/L and 5.8 (95% CI 2.9–8.7) for 140 μg/L [63]. Detoxification of arsenic may occur through a methylation pathway. The organic methylated arsenicals are much less potent as mutagenic agents than the inorganic arsenicals [90]. Several epidemiological studies suggested that the exposure to disinfection byproducts of chlorinated water such as trihalomethanes [91] and nitrosamines [92] was associated with bladder cancer. There are other potential candidates of bladder carcinogen in chlorinated drinking water, haloamides, halocyclopentenoic acids, furans, and haloquinones [93]. Further studies are needed to determine the causal agents in chlorinated drinking water.

Li F. et al. found an increased risk of bladder cancer associated with processed meat (RR 1.22, 95% CI 1.04–1.43) but not with red meat (RR 1.15, 95% CI 0.97–1.36) in a meta-analysis [94]. Processed meat may produce a carcinogen related to inflammation. Several meta-analyses demonstrated that dietary intake of vitamin A, C, D, and E could reduce a risk of bladder cancer, respectively [13,14,63,95].

### 3.5. Metabolic Syndrome

In a case-control study with 690 incident bladder cancer patients and 665 cancer-free matched patients, metabolic syndrome was at a twofold higher risk of bladder cancer (95% CI 1.38–3.19) [96]. In a retrospective analysis of 169 patients who underwent transurethral resection, metabolic syndrome was significantly associated with a high histological grade [97]. No association between metabolic syndrome and risk of NMIBC recurrence was found in a cohort of 1485 older (age ≥ 60 years) NMIBC patients [98].

Metabolic syndrome is defined as the presence of three of the following: Hypertension, hyperlipidemia, diabetes, or body mass index >30. Cantiello F. et al. revealed that obesity suggests a possible correlation with pathological factors and prognosis of bladder cancer and that diabetes seems to be associated with worse oncological outcomes [99]. Diabetes also showed a significantly higher relative risk (1.36–1.51) after adjustment for age, sex, and other potential cofounders [100]. Hyperlipidemia exhibited a 37–51% increased risk of bladder cancer compared with nonhyperlipidemia [101].

### 3.6. Others

Cyclophosphamide, an alkylating agent, and pioglitazone, an antidiabetic drug of the thiazolidinedione class, have been suggested to have a relation to bladder cancer incidence with long-term use [102].

A large cohort study demonstrated a reduced risk of bladder cancer with the use of oral antidiabetic agents, metformin and sulphonylurea [103]. Another cohort study found no association of insulin with bladder cancer risk [104].

Radiotherapy to treat various cancers located in the pelvic region, especially prostate cancer, has been associated with developing second malignancies of the bladder. In a meta-analysis, bladder cancer risk was elevated with a hazard ratio of 1.67 (95% CI 1.55–1.80) after radiation of prostate [105]. Histologically, the number of non-urothelial cell carcinoma was greater than usual, and CIS was more common [106]. Anatomically, tumors were more frequently found at the trigone.

## 4. Conclusions

Comprehensive analyses revealed mutational landscape of bladder cancer. Further investigations for the function of each mutation are warranted. Cigarette smoking, exposures to chemicals and gases, certain medications, radiation, and genetic factors are established risk factors for bladder cancer. Diet, nutrition, and metabolic syndrome, which are linked to lifestyle, are also suggested to be risk factors for bladder cancer. Identifying the relationship between the mutations and the lifestyle could be useful for prevention and treatment of bladder cancer.

## References

[1] Antoni S., Ferlay J., Soerjomataram I., Znaor A., Jemal A., Bray F. (2017). Bladder cancer incidence and mortality: A global overview and recent trends. Eur. Urol..

[2] Prout G.R.Jr., Barton B.A., Griffin P.P., Friedel G.H. (1992). Treated history of noninvasive grade 1 transitional cell carcinoma. The National Bladder Cancer Group. J. Urol..

[3] Sanli O., Dobruch J., Knowles M.A., Burger M., Alemozaffar M., Nielsen M.E., Lotan Y. (2017). Bladder cancer. Nat. Rev. Dis. Primers..

[4] Nakayama M., Ito Y., Hatano K., Nakai Y., Kakimoto K., Miyashiro I., Nishimura K. (2019). Impact of sex difference on survival of bladder cancer: A population-based registry data in Japan. Int. J. Urol..

[5] Hurst C.D., Knowles M.A. (2018). Mutational landscape of non-muscle-invasive bladder cancer. Urol. Oncol..

[6] Sjödahl G., Lauss M., Lövgren K., Chebil G., Gudjonsson S., Veerla S., Patschan O., Aine M., Fernö M., Ringnér M. (2012). A molecular taxonomy for urothelial carcinoma. Clin. Cancer Res..

[7] Choi W., Porten S., Kim S., Willis D., Plimack E.R., Hoffman-Censits J., Roth B., Cheng T., Tran M., Lee I.-L. (2014). Identification of distinct basal and luminal subtypes of muscle-invasive bladder cancer with different sensitivities to frontline chemotherapy. Cancer Cell..

[8] Cancer Genome Atlas Research Network (2014). Comprehensive molecular characterization of urothelial bladder carcinoma. Nature.

[9] Damrauer J.S., Hoadley K.A., Chism D.D., Fan C., Tiganelli C.J., Wobker S.E., Yeh J.J., Milowsky M.I., Iyer G., Parker J.S. (2014). Intrinsic subtypes of high-grade bladder cancer reflect the hallmarks of breast cancer biology. Proc. Natl. Acad. Sci. USA.

[10] Knowles M.A., Hurst C.D. (2015). Molecular biology of bladder cancer: New insights into pathogenesis and clinical diversity. Nat. Rev. Cancer.

[11] McConkey D.J., Choi W., Dinney C.P. (2015). Genetic subtypes of invasive bladder cancer. Curr. Opin. Urol..

[12] McConkey D.J., Choi W., Marquis L., Martin F., Williams M.B., Shah J., Svatek R., Das A., Adam L., Kamat A. (2009). Role of epithelial-to-mesenchymal transition (EMT) in drug sensitivity and metastasis in bladder cancer. Cancer Metastasis Rev..

[13] Al-Zalabani A.H., Stewart K.F., Wesselius A., Schols A.M., Zeegers M.P. (2016). Modifiable risk factors for the prevention of bladder cancer: A systematic review of meta-analyses. Eur. J. Epidemiol..

[14] Mostafid H., Fankhauser C.D. (2018). Prevention of bladder cancer incidence and recurrence: Nutrition and lifestyle. Curr. Opin. Urol..

[15] Ruiz-Núñez B., Pruimboom L., Dijck-Brouwer D.A., Muskiet F.A. (2013). Lifestyle and nutritional imbalances associated with Western diseases: Causes and consequences of chronic systemic low-grade inflammation in an evolutionary context. J. Nutr. Biochem..

[16] Hayashi T., Fujita K., Matsushita M., Nonomura N. (2019). Main inflammatory cells and potentials of anti-inflammatory agents in prostate cancer. Cancers (Basel).

[17] Lu D.L., Ren Z.J., Zhang Q., Ren P.W., Yang B., Liu L.R., Dong Q. (2018). Meta-analysis of the association between the inflammatory potential of diet and urologic cancer risk. PLoS One.

[18] Lee H.W., Park S.H., Weng M.W., Wang H.T., Huang W.C., Lepor H., Wu X.R., Chen L.C., Tang M.S. (2018). E-cigarette smoke damages DNA and reduces repair activity in mouse lung, heart, and bladder as well as in human lung and bladder cells. Proc. Natl. Acad. Sci. USA.

[19] Kinde I., Munari E., Faraj S.F., Hruban R.H., Schoenberg M., Bivalacqua T., Allaf M., Springer S., Wang Y., Diaz L.A.Jr. (2013). TERT promoter mutations occur early in urothelial neoplasia and are biomarkers of early disease and disease recurrence in urine. Cancer Res..

[20] Leão R., Lee D., Figueiredo A., Hermanns T., Wild P., Komosa M., Lau I., Mistry M., Nunes N.M., Price A.J. (2019). Combined genetic and epigenetic alterations of the TERT promoter affect clinical and biological behavior of bladder cancer. Int. J. Cancer..

[21] Pena M.D.C.R., Tregnago A.C., Eich M.-L., Springer S., Wang Y., Taheri D., Ertoy D., Fujita K., Bezerra S.M., Cunha I.W. (2017). Spectrum of genetic mutations in de novo PUNLMP of the urinary bladder. Virchows Arch..

[22] Nguyen D., Taheri D., Springer S., Cowan M., Guner G., Rodriguez M.A.M., Wang Y., Kinde I., VandenBussche C.J., Olson M.T. (2016). High prevalence of TERT promoter mutations in micropapillary urothelial carcinoma. Virchows Arch..

[23] Palsgrove D.N., Taheri D., Springer S.U., Cowan M., Guner G., Rodriguez M.A.M., Pena M.D.C.R., Wang Y., Kinde I., Ricardo B.F.P. (2019). Targeted sequencing of plasmacytoid urothelial carcinoma reveals frequent TERT promoter mutations. Hum. Pathol..

[24] Cowan M.L., Springer S., Nguyen D., Taheri D., Guner G., Rodriguez M.A.M., Wang Y., Kinde I., Pena M.D.C.R., VandenBussche C.J. (2016). Detection of TERT promoter mutations in primary adenocarcinoma of the urinary bladder. Hum. Pathol..

[25] Cowan M., Springer S., Nguyen D., Taheri D., Guner G., Rodriguez M.A.M., Wang Y., Kinde I., VandenBussche C.J., Olson M.T. (2016). High prevalence of TERT promoter mutations in primary squamous cell carcinoma of the urinary bladder. Mod. Pathol..

[26] Hayashi Y., Fujita K., Nojima S., Tomiyama E., Matsushita M., Koh Y., Nakano K., Wang C., Ishizuya Y., Kato T. (2020). *TERT* C228T mutation in non-malignant bladder urothelium is associated with intravesical recurrence for patients with non-muscle invasive bladder cancer. Mol. Oncol..

[27] Springer S.U., Chen C.-H., Pena M.D.C.R., Li L., Douville C., Wang Y., Cohen J.D., Taheri D., Silliman N., Schaefer J. (2018). Non-invasive detection of urothelial cancer through the analysis of driver gene mutations and aneuploidy. Elife.

[28] Hayashi Y., Fujita K., Matsuzaki K., Matsushita M., Matsushita M., Kawamura N., Koh Y., Nakano K., Wang C., Ishizuya Y. (2019). Diagnostic potential of TERT promoter and FGFR3 mutations in urinary cell-free DNA in upper tract urothelial carcinoma. Cancer Sci..

[29] Eich M.-L., Pena M.D.C.R., Springer S.U., Taheri D., Tregnago A.C., Salles D.C., Bezerra S.M., Cunha I.W., Fujita K., Ertoy D. (2019). Incidence and distribution of UroSEEK gene panel in a multi-institutional cohort of bladder urothelial carcinoma. Mod. Pathol..

[30] Hayashi Y., Fujita K., Matsuzaki K., Eich M.-L., Tomiyama E., Matsushita M., Koh Y., Nakano K., Wang C., Ishizuya Y. (2020). Clinical significance of hotspot mutation analysis of urinary cell-free DNA in urothelial bladder cancer. Front. Oncol..

[31] Robertson A.G., Kim J., Al-Ahmadie H., Bellmunt J., Guo G., Cherniack A.D., Hinoue T., Laird P.W., Hoadley K.A., Akbani R. (2017). Comprehensive molecular characterization of muscle-invasive bladder cancer. Cell.

[32] Williams S.V., Hurst C.D., Knowles M.A. (2013). Oncogenic FGFR3 gene fusions in bladder cancer. Hum. Mol. Genet..

[33] Casadei C., Dizman N., Schepisi G., Cursano M.C., Basso U., Santini D., Pal S.K., De Giorgi U. (2019). Targeted therapies for advanced bladder cancer: New strategies with FGFR inhibitors. Ther. Adv. Med. Oncol..

[34] Loriot Y., Necchi A., Park S.H., Garcia-Donas J., Huddart R., Burgess E., Fleming M., Rezazadeh A., Mellado B., Varlamov S. (2019). Erdafitinib in locally advanced or metastatic urothelial carcinoma. N Engl. J. Med..

[35] Oxford G., Theodorescu D. (2003). The role of Ras superfamily proteins in bladder cancer progression. J. Urol..

[36] Dangle P.P., Zaharieva B., Jia H., Pohar K.S. (2009). Ras-MAPK pathway as a therapeutic target in cancer – emphasis on bladder cancer. Recent Pat. Anticancer Drug Discov..

[37] Knowles M.A., Platt F.M., Ross R.L., Hurst C.D. (2009). Phosphatidylinositol 3-kinase (PI3K) pathway activation in bladder cancer. Cancer Metastasis Rev..

[38] Schulz W.A., Lang A., Koch J., Greife A. (2019). The histon demethylase UTX/KDM6A in cancer: Progress and puzzles. Int J. Cancer.

[39] Hurst C.D., Alder O., Platt F.M., Droop A., Stead L.F., Burns J.E., Burghel G.J., Jain S., Klimczak L.J., Lindsay H. (2017). Genomic subtypes of non-invasive bladder cancer with distinct metabolic profile and female gender bias in KDM6A mutation frequency. Cancer Cell..

[40] Ler L.D., Ghosh S., Chai X., Thike A.A., Heng H.L., Siew E.Y., Dey S., Koh L.K., Lim J.Q., Lim W.K. (2017). Loss of tumor suppressor KDM6A amplifies PRC-regulated transcriptional repression in bladder cancer and can be targeted through inhibition of EZH2. Sci. Transl. Med..

[41] Kobatake K., Ikeda K., Nakata Y., Yamasaki N., Ueda T., Kanai A., Sentani K., Sera Y., Hayashi T., Koizumi M. (2020). *Kdm6a* deficiency activates inflammatory pathways, promotes M2 macrophage polarization, and causes bladder cancer in cooperation with *p53* dysfunction. Clin. Cancer Res..

[42] Pymar L.S., Platt F.M., Askham J.M., Morrison E.E., Knowles M.A. (2008). Bladder tumour-derived somatic TSC1 missense mutations cause loss of function via distinct mechanisms. Hum. Mol. Genet..

[43] Guo Y., Chekaluk Y., Zhang J., Du J., Gray N.S., Wu C.-L., Kwiatkowski D.J. (2013). TSC1 involvement in bladder cancer: Diverse effects and therapeutic implications. J. Pathol..

[44] Woodford M.R., Hughes M., Sager R.A., Backe S.J., Baker-Williams A.J., Bratslavsky M.S., Jacob J.M., Shapiro O., Wong M., Bratslavsky G. (2019). Mutation of the co-chaperone Tsc1 in bladder cancer diminishes Hsp90 acetylation and reduces drug sensitivity and selectivity. Oncotarget..

[45] Rebouissou S., Hérault A., Letouzé E., Neuzillet Y., Laplanche A., Ofualuka K., Maillé P., Leroy K., Riou A., Lepage M.L. (2012). CDKN2A homozygous deletion is associated with muscle invasion in FGFR3-mutated urothelial bladder carcinoma. J. Pathol..

[46] Worst T., Weis C.-A., Stöhr R., Bertz S., Eckstein M., Otto W., Breyer J., Hartmann A., Bolenz C., Wirtz R.M. (2018). CDKN2A as transcriptomatic marker for muscle-invasive bladder cancer risk stratification and therapy decision-making. Sci. Rep..

[47] van Rhijn B.W., van der Kwast T.H., Vis A.N., Kirkels W.J., Boevé E.R., Jöbsis A.C., Zwarthoff E.C. (2004). FGFR3 and P53 characterize alternative genetic pathways in the pathogenesis of urothelial cell carcinoma. Cancer Res..

[48] Ciccarese C., Massari F., Blanca A., Tortora G., Montironi R., Cheng L., Scarpelli F., Raspollini M.R., Vau N., Fonseca J. (2017). Tp53 and its potential therapeutic role as a target in bladder cancer. Expert Opin. The. Target..

[49] Delker D.A., Yano B.L., Gollapudi B.B. (2000). Evaluation of cytotoxicity, cell proliferation, and genotoxicity induced by *p*-cresidine in hetero- and nullizygous transgenic p53 mice. Toxicol. Sci..

[50] Li Q., Damish A.W., Frazier Z., Liu D., Reznichenko E., Kamburov A., Bell A., Zhao H., Jordan E.J., Gao S.P. (2019). *ERCC2* helicase domain mutations confer nucleotide excision repair deficiency and drive cisplatin sensitivity in muscle-invasive bladder cancer. Clin. Cancer Res..

[51] Van Allen E.M., Mouw K.W., Kim P., Iyer G., Wagle N., Al-Ahmadie H.A., Zhu C., Ostrovnaya I., Kryukov G.V., O’Connor K.W. (2014). Somatic ERCC2 mutations correlate with cisplatin sensitivity in muscle-invasive urothelial carcinoma. Cancer Discov..

[52] Liu D., Plimack E.R., Hoffman-Censits J., Garraway L.A., Bellmunt J., Van Allen E., Rosenberg J.E. (2016). Clinical validation of chemotherapy response biomarker ERCC2 in muscle-invasive urothelial bladder carcinoma. JAMA Oncol..

[53] Feki-Tounsi M., Khlifi R., Louati I., Fourati M., Mhiri M.-N., Hamza-Chaffai A., Rebai A. (2017). Polymorphisms in XRCC1, ERCC2, and ERCC3 DNA repair genes, CYP1A1 xenobiotic metabolism gene, and tobacco are associated with bladder cancer susceptibility in Tunisian population. Environ. Sci. Pollut. Res. Int..

[54] Solomon D.A., Kim J.-S., Bondaruk J., Shariat S.F., Wang Z.-F., Elkahloun A.G., Ozawa T., Gerard J., Zhuang D., Zhang S. (2013). Frequent truncating mutations of STAG2 in bladder cancer. Nat. Genet..

[55] Osei-Amponsa V., Buckwalter J.M., Shuman L., Zheng Z., Yamashita H., Walter V., Wildermuth T., Ellis-Mohl J., Liu C., Warrick J.I. (2020). Hypermethylation of *FOXA1* and allelic loss of *PTEN* drive squamous differentiation and promote heterogeneity in bladder cancer. Oncogene.

[56] Ploeg M., Aben K.K., Kiemeney L.A. (2009). The present and future burden of urinary bladder cancer in the world. World J. Urol..

[57] Fantini D., Seiler R., Meeks J.J. (2019). Molecular footprints of muscle-invasive bladder cancer in smoking and nonsmoking patients. Urol. Oncol..

[58] Saito R., Smith C.C., Utsumi T., Bixby L.M., Kardos J., Wobker S.E., Stewart K.G., Chai S., Manocha U., Byrd K.M. (2018). Molecular subtype-specific immunocompetent models of high-grade urothelial carcinoma reveal differential neoantigen expression and response to immunotherapy. Cancer Res..

[59] Bourn J., Rathore K., Donnell R., White W., Uddin M.J., Marnett L., Cekanova M. (2019). Detection of carcinogen-induced bladder cancer by fluorocoxib A. BMC Cancer..

[60] Yu D., Geng H., Liu Z., Zhao L., Liang Z., Zhang Z., Xie D., Wang Y., Zhang T., Min J. (2017). Cigarette smoke induced urocytic epithelial mesenchymal transition via MAPK pathways. Oncotarget.

[61] Sun X., Deng Q., Liang Z., Liu Z., Geng H., Zhao L., Zhou Q., Liu J., Ma J., Wang D. (2017). Cigarette smoke extract induces epithelial-to-mesenchymal transition of human bladder cancer T24 cells through activation of ERK1/2 pathway. Biomed. Pharmacother..

[62] Kispert S., Marentette J., McHowat J. (2019). Cigarette smoking promotes bladder cancer via increased platelet-activating factor. Physiol. Rep..

[63] Cumberbatch M.G.K., Jubber I., Black P.C., Esperto F., Figueroa J.D., Kamat A.M., Kiemeney L., Lotan Y., Pang K., Silverman D.T. (2018). Epidemiology of bladder cancer: A systematic review and contemporary update of risk factors in 2018. Eur. Urol..

[64] Kirkland D., Aardema M., Henderson L., Müller L. (2005). Evaluation of the ability of a battery of three in vitro genotoxicity tests to discriminate rodent carcinogens and non-carcinogens I. Sensitivity, specificity and relative predictivity. Mutat. Res..

[65] Kassie F., Knasmüller S. (2000). Genotoxic effects of allyl isothiocyanate (AITC) and phenethyl isothiocynate (PEITC). Chem. Biol. Interact..

[66] Vermeulen S.H., Hanum N., Grotenhuis A.J., Castaño-Vinyals G., van der Heijden A.G., Aben K.K., Mysorekar I.U., Kiemeney L.A. (2015). Recurrent urinary tract infection and risk of bladder cancer in the Nijimegen bladder cancer. Br. J. Cancer..

[67] Kassouf W., Spiess P.E., Siefker-Radtke A., Swanson D., Grossman H.B., Kamat A.M., Munsell M.F., Guo C.C., Czerniak B.A., Dinney C.P. (2007). Outcome and patterns of recurrence of non-bilharzial pure squamous cell carcinoma of bladder. Cancer.

[68] Adebayo A.S., Suryavanshi M.V., Bhute S., Agunloye A.M., Isokpehi R.D., Anumudu C.I., Shouche Y.S. (2017). The microbiome in urogenital schistosomiasis and induced bladder pathologies. PLOS Negl. Trop. Dis..

[69] Mostafa M.H., Sheweita S.A., O’Connor P.J. (1999). Relationship between schistosomiasis and bladder cancer. Clin. Microbiol. Rev..

[70] Alfano M., Canducci F., Nebuloni M., Clementi M., Montorsi F., Salonia A. (2017). The interplay of extracellular matrix and microbiome in urothelial bladder cancer. Nat. Rev. Cancer..

[71] Markowski M.C., Boorjian S.A., Burton J.P., Hahn N.M., Ingersoll M.A., Vareki S.M., Pal S.K., Sfanos K.S. (2019). The microbiome and genitourinary cancer: A collaborative review. Eur. Urol..

[72] Shivappa N., Hébert J.R., Rosato V., Rossi M., Libra M., Montella M., Serraino D., La Vecchia C. (2017). Dietary inflammatory index and risk of bladder cancer in a large Itarian case-control study. Urology.

[73] Dugué P.A., Hodge A.M., Brinkman M.T., Bassett J.K., Shivappa N., Hébert J.R., Hopper J.L., English D.R., Milne R.L., Giles G.G. (2016). Association between selected dietary scores and the risk of urothelial cell carcinoma: A prospective cohort study. Int. J. Cancer..

[74] Abufaraj M., Tabung F.K., Shariot S.F., Moschini M., Devore E., Parantoniou K., Yang L., Strohmaier S., Rohrer F., Markt S.C. (2019). Association between inflammatory potential diet and bladder cancer risk: Results of 3 United States prospective cohort study. J. Urol..

[75] Luo J., Shivappa N., Hébert J.R., Xu X. (2020). Dietary inflammatory index and bladder cancer risk: A prospective study. Eur. J. Clin. Nutr..

[76] Hu J., La Vecchia C., de Groh M., Negri E., Morrison H., Mery L., the Canadian Cancer Registries Epidemiology Research Group (2012). Dietary cholesterol intake and cancer. Ann. Oncol..

[77] Brinkman M.T., Karagas M.R., Zens M.S., Schned A.R., Reulen R.C., Zeegers M.P. (2011). Intake of α-linolenic acid and other fatty acids in relation to the risk of bladder cancer: Results from New Hampshire case-control study. Br. J. Nutr..

[78] Yao B., Yan Y., Ye X., Fang H., Xu H., Liu Y., Li S., Zhao Y. (2014). Intake of fruit and vegetables and risk of bladder cancer: A dose-respense meta-analysis of observational studies. Cancer Causes Control.

[79] Liu H., Wang X.-C., Hu G.-H., Guo Z.-F., Lai P., Xu L., Huang T.-B., Xu Y.-F. (2015). Fruit and vegetable consumption and risk of bladder cancer: An updated meta-analysis of observational studies. Eur. J. Cancer Prev..

[80] Lin J., Kamat A., Gu J., Chen M., Dinney C.P., Forman M.R., Wu X. (2009). Dietary intake of vegetables and fruits and the modification effects of GSTM1 and NAT2 genotypes on bladder cancer risk. Cancer Epidemiol. Biomark. Prev..

[81] Fragoso M.F., Prado M.G., Barbosa L., Rocha N.S., Barbisan L.F. (2012). Inhibition of mouse urinary bladder carcinogenesis by açai friut (Euterpe Oleraceae Martius) intake. Plant. Foods Hum. Nutr..

[82] Zhou Y., Tian C., Jia C. (2012). A dose-response meta-analysis of coffee consumption and bladder cancer. Prev. Med..

[83] Wu W., Tong Y., Zhao Q., Yu G., Wei X., Lu Q. (2015). Coffee consumption and bladder cancer: A meta-analysis of observational studies. Sci. Rep..

[84] Loftfield E., Freedman N.D., Inoue-Choi M., Graubard B.I., Sinha R. (2017). A prospective investigation of coffee drinking and bladder cancer incidence in the United States. Epidemiology.

[85] Yu X., Bao Z., Zou J., Dong J. (2011). Coffee consumption and risk of bladder cancers: A meta-analysis of cohort studies. BMC Cancer..

[86] Sugiyama K., Sugawara Y., Tomata Y., Nishino Y., Fukao A., Tsuji I. (2017). The association between coffee consumption and bladder cancer incidence in a pooled analysis of the Miyagi Cohort Study and Ohsaki Cohort Study. Eur. J. Cancer Prev..

[87] Dai Z.-W., Cai K.-D., Li F.-R., Wu X.-B., Chenm G.-C. (2019). Association between coffee consumption and risk of bladder cancer in a meta-analysis of 16 prospective studies. Nutr. Metab. (Lond).

[88] Yu E.Y.W., Wesselius A., van Osch F., Stern M.C., Jiang X., Kellen E., Lu C.-M., Pohlabeln H., Steineck G., Marshall J. (2019). The association of coffee consumption and bladder cancer in the bladder cancer epidemiology and nutritional determinants (BLEND) international pooled study. Cancer Causes Control.

[89] Yu E.Y.W., Dai Y., Wesselius A., van Osch F., Brinkman M., van den Brandt P., Grant E.J., White E., Weiderpass E., Gunter M. (2020). Coffee consumption and risk of bladder cancer: A pooled analysis of 501,604 participants from 12 cohort studies in the Bladder Cancer Epidemiology and Nutritional Determinants (BLEND) international study. Eur. J. Epidemiol..

[90] Moore M.M., Harrington-Brock K., Doerr C.L. (1997). Relative genotoxic potency of arsenic and its methylated metabolites. Mutat. Res..

[91] Costet N., Villanueva C.M., Jaakkola J.J.K., Kogevinas M., Cantor K.P., King W.D., Lynch C.F., Nieuwenhuijsen M.J., Cordier S. (2011). Water disinfection by-products and bladder cancer: Is there a European specificity? A pooled and meta-analysis of European case-control studies. Occup. Environ. Med..

[92] Barry K.H., Jones R.R., Cantor K.P., Freeman L.E.B., Wheeler D.C., Baris D., Johnson A.T., Hosain G.M., Schwenn M., Zhang H. (2020). Ingested nitrate and nitrite and bladder cancer in Northern New England. Epidemiology.

[93] Diana M., Felipe-Sotelo M., Bond T. (2019). Disinfection byproducts potentially responsible for the association between chlorinated drinking water and bladder cancer: A review. Water Res..

[94] Li F., An S., Hou L., Chen P., Lei C., Tan W. (2014). Red and processed meat intake and risk of bladder cancer: A meta-analysis. Int. J. Clin. Exp. Med..

[95] Piyathilake C. (2016). Dietary factors associated with bladder cancer. Investig. Clin. Urol..

[96] Montella M., Di Maso M., Crispo A., Grimaldi M., Bosetti C., Tutati F., Giudice A., Libra M., Serraino D., La Vecchia C. (2015). Metabolic syndrome and the risk of urothelial carcinoma of the bladder: A case-control study. BMC Cancer.

[97] Nagase K., Tobu S., Kusano S., Takahara K., Udo K., Noguchi M. (2018). The association between metabolic syndrome and high-stage primary urothelial carcinoma of the bladder. Curr. Urol..

[98] Garg T., Young A.J., O’Keeffe-Rosetti M., McMullen C.K., Nielsen M.E., Murphy T.E., Kirchner H.L. (2020). Association between metabolic syndrome and recurrence of nonmuscle-invasive bladder cancer in older adults. Urol. Oncol..

[99] Cantiello F., Cicione A., Salonia A., Autorio R., De Nunzio C., Briganti A., Gandaglia G., Dell’Oglio P., Capogrosso P., Damiano R. (2015). Association between metabolic syndrome, obesity, diabetes millitus and oncological outcomes of bladder cancer: A systematic review. Int. J. Urol..

[100] Tseng C.H. (2011). Diabetes and risk of bladder cancer: A study using National Health Insurance database in Taiwan. Diabetologia.

[101] Radisauskas R., Kuzmickiene I., Milinaviciene E., Everatt R. (2016). Hypertension, serum lipids and cancer risk: A review of epidemiological evidence. Medicina.

[102] Burger M., Catto J.W., Dalbagni G., Grossman H.B., Herr H., Karakiewicz P., Kassouf W., Kiemeney L.A., La Vecchia C., Shariat S. (2013). Epidemiology and risk factors of urothelial bladder cancer. Eur. Urol..

[103] Tseng C.-H. (2014). Metformin may reduce bladder cancer risk in Taiwanese patients with type 2 diabetes. Acta Diabetol..

[104] Tseng C.-H. (2014). Human insulin does not increase bladder cancer risk. PLoS One.

[105] Wallis C.J.D., Mahar A.L., Choo R., Herschorn S., Kodama R.T., Shah P.S., Danjoux C., Narod S.A., Nam R.K. (2016). Second malignancies after radiotherapy for prostate cancer: Systematic review and meta-analysis. BMJ.

[106] Abern M.R., Dude A.M., Tsivian M., Coogan C.L. (2013). The characteristics of bladder cancer after radiotherapy for prostate cancer. Urol. Oncol..

